# On the Achievable Throughput Over TVWS Sensor Networks

**DOI:** 10.3390/s16040457

**Published:** 2016-03-30

**Authors:** Marcello Caleffi, Angela Sara Cacciapuoti

**Affiliations:** 1 Department of Electrical Engineering and Information Technologies, University of Naples Federico II, Naples 80126, Italy; angelasara.cacciapuoti@unina.it; 2 Multimedia Communications Laboratory, CNIT, Naples 80126, Italy

**Keywords:** white space, secondary, cognitive radio, sensor network, J0101

## Abstract

In this letter, we study the throughput achievable by an unlicensed sensor network operating over TV white space spectrum in presence of coexistence interference. Through the letter, we first analytically derive the achievable throughput as a function of the channel ordering. Then, we show that the problem of deriving the maximum expected throughput through exhaustive search is computationally unfeasible. Finally, we derive a computational-efficient algorithm characterized by polynomial-time complexity to compute the channel set maximizing the expected throughput and, stemming from this, we derive a closed-form expression of the maximum expected throughput. Numerical simulations validate the theoretical analysis.

## 1. Introduction

In recent years, regulatory bodies such as FCC and Ofcom [1,2,3] have approved the dynamic access of unlicensed sensor networks, referred to in the following as Secondary Sensor Networks (SSNs), to the TV White Space (TVWS) spectrum. The existing regulations circumvented the need for sensing algorithms for establishing the availability of free TVWS spectrum [4,5,6,7], since they require the SSNs to periodically obtain the the list of TVWS channels free from licensed users from a geolocated database [8,9]. Hence, it is reasonable to expect that multiple and heterogeneous SSNs based on different standards such as [10,11,12] are expected to coexist within the same geographical region over shared TVWS spectrum. Stemming from this, in this paper we develop an analytical framework for modeling the throughput achievable by an arbitrary SSN operating over shared TVWS spectrum in presence of coexistence interference. Specifically, we first analytically derive the expected throughput as a function of the channel ordering. Then, the problem of deriving the maximum expected throughput through exhaustive search is shown to be computationally unfeasible. Furthermore, a computational-efficient algorithm for ordering the channels to maximize the expected throughput is designed and, stemming from this, a closed-form expression of the maximum expected throughput is derived. Finally, numerical results validate the theoretical analysis.

## 2. System Model

We consider a SSN communicating through the TVWS spectrum in agreement with the existing standards and regulations. Hence, with an access to the TVWS database, the SSN obtains the list of channels free from licensed users. In the following, we denote with N={1,2,…,N} the set of free channels, and with $T_{i}$ the expected throughput for the *i*-th channel. For the reasons described in the introduction, any incumbent-free channel in N may be affected by coexisting interference caused by other heterogenous SSNs operating over the same TVWS channel within the same geographical area. In the following, $p_{i}$ denotes the probability of the *i*-th channel being not affected by coexistence interference (the SSN can estimate the interference probability through the past interference history [8]), with ${\bar{p}}_{i}\overset{▵}{=}1-p_{i}$. Furthermore, C denotes an arbitrary channel set, *i.e.*, an ordered sequence of available channels without repetition:

(1) $$C=\{\left(c_{\alpha_{1}},c_{\alpha_{2}},\ldots ,c_{\alpha_{N}}\right):c_{\alpha_{i}}\neq c_{\alpha_{j}}\wedge \alpha_{i}\in N\}$$

The order among the channels reflects the priorities for channel utilization, *i.e.*, channel $c_{\alpha_{i}}$ is used if and only if: (i) all the channels with higher priority $c_{\alpha_{1}},\ldots ,c_{\alpha_{i-1}}$ are affected by coexistence interference; (ii) channel $c_{\alpha_{i}}$ is not affected by coexistence interference. In the following, we denote with $C^{*}$ the *optimal* channel set, *i.e.*, the channel set that maximizes the expected throughput.

## 3. Expected Throughput

In this section, we first derive the closed-form expression of the expected throughput as a function of the adopted channel set, say $C=(c_{\alpha_{1}},c_{\alpha_{2}},\ldots ,c_{\alpha_{N}})$. To this aim, some preliminaries are needed. We denote with $q_{c_{\alpha_{i}}}$ the probability that the first i-1 channels $c_{\alpha_{1}},\ldots ,c_{\alpha_{i-1}}$ with higher priority with respect to $c_{\alpha_{i}}$ are affected by coexistence interference. By exploiting the reasonable hypothesis of independent SSN activities over different channels, it results that $q_{c_{\alpha_{i}}}$ is given by:

(2) $$q_{c_{\alpha_{i}}}=\prod_{k=1}^{i-1}{\bar{p}}_{c_{\alpha_{k}}}$$

Hence, the expected achievable throughput ${\bar{T}}_{C}$ is equal to:

(3) $${\bar{T}}_{C}=\sum_{i=1}^{N}p_{c_{\alpha_{i}}}\left(\prod_{k=1}^{i-1}{\bar{p}}_{c_{\alpha_{k}}}\right)T_{c_{\alpha_{i}}}=\sum_{i=1}^{N}p_{c_{\alpha_{i}}}q_{c_{\alpha_{i}}}T_{c_{\alpha_{i}}}$$

with $p_{c_{\alpha_{i}}}$ denoting the probability of channel $c_{\alpha_{i}}$ being not affected by coexistence interference and $T_{c_{\alpha_{i}}}$ denoting channel $c_{\alpha_{i}}$ expected throughput, respectively. From Equation (3), it results that ${\bar{T}}_{C}$ depends on the adopted channel set. In the following, by deriving the optimal channel set $C^{*}$, we are able to compute the maximum expected throughput ${\bar{T}}^{*}$ achievable by an arbitrary SSN operating over shared TVWS spectrum:

(4) $${\bar{T}}^{*}=\max_{C}\left\{{\bar{T}}_{C}\right\}$$

We first observe that the computation of the maximum throughput ${\bar{T}}^{*}$ through exhaustive search is computationally unfeasible. In fact, the number of channel sets is equal to the number N! of permutations of N=|N| distinct objects. Consequently, computing ${\bar{T}}^{*}$ via exhaustive search is as much computational hard as solving the NP-hard *traveling salesman problem* via brute-force search. Nevertheless, in the following, we derive a rule for ordering the channels to maximize the expected throughput and, stemming from this result, we design a computational feasible algorithm for computing ${\bar{T}}^{*}$.

## 4. Maximum Expected Throughput

Let us suppose that $T_{c_{\alpha_{m}}}>T_{c_{\alpha_{m+1}}}$. We prove that channel $c_{\alpha_{m}}$ must have higher priority than channel $c_{\alpha_{m+1}}$ with a *reductio ad absurdum* by supposing that there exist a channel set $C^{\prime }$ different by channel set $C=(c_{\alpha_{1}},c_{\alpha_{2}},\ldots ,c_{\alpha_{N}})$ defined as:

(5) $$C^{\prime }=\left(c_{\alpha_{1}}^{\prime },c_{\alpha_{2}}^{\prime },\ldots ,c_{\alpha_{N}}^{\prime }\right):\left\{\begin{array}{c} c_{\alpha_{i}}^{\prime }=c_{\alpha_{i}}\;\forall \;i\neq m,m+1\\ c_{\alpha_{m}}^{\prime }=c_{\alpha_{m+1}}\\ c_{\alpha_{m+1}}^{\prime }=c_{\alpha_{m}} \end{array}\right.$$

so that:

(6) $${\bar{T}}_{C}=\sum_{i=1}^{N}p_{c_{\alpha_{i}}}q_{c_{\alpha_{i}}}T_{c_{\alpha_{i}}}<{\bar{T}}_{C^{\prime }}=\sum_{i=1}^{N}p_{c_{\alpha_{i}}^{\prime }}q_{c_{\alpha_{i}}^{\prime }}T_{c_{\alpha_{i}}^{\prime }}$$

By accounting for Equation (2), we have

(7) $$q_{c_{\alpha_{i}}^{\prime }}=\prod_{k=1}^{i-1}{\bar{p}}_{c_{\alpha_{k}}^{\prime }}=\prod_{k=1}^{i-1}{\bar{p}}_{c_{\alpha_{k}}}=q_{c_{\alpha_{i}}}\;\forall i\leq m$$

As a consequence, by using Equations (5) and (6), it results:

(8) $$\begin{array}{cc} q_{c_{\alpha_{m+1}}^{\prime }} & =\prod_{k=1}^{m}{\bar{p}}_{c_{\alpha_{k}}^{\prime }}={\bar{p}}_{c_{\alpha_{m}}^{\prime }}\prod_{k=1}^{m-1}{\bar{p}}_{c_{\alpha_{k}}}={\bar{p}}_{c_{\alpha_{m+1}}}q_{c_{\alpha_{m}}} \end{array}$$

(9) $$\begin{array}{cc} q_{c_{\alpha_{m+2}}^{\prime }} & =\prod_{k=1}^{m+1}{\bar{p}}_{c_{\alpha_{k}}^{\prime }}={\bar{p}}_{c_{\alpha_{m+1}}^{\prime }}{\bar{p}}_{c_{\alpha_{m+1}}}q_{c_{\alpha_{m}}}=q_{c_{\alpha_{m+2}}} \end{array}$$

By accounting for Equations (7) and (9), we have $p_{c_{\alpha_{i}}}q_{c_{\alpha_{i}}}T_{c_{\alpha_{i}}}=p_{c_{\alpha_{i}}^{\prime }}q_{c_{\alpha_{i}}^{\prime }}T_{c_{\alpha_{i}}^{\prime }}$ for any i≠m,m+1. Hence, Equation (6) is equivalent to:

(10) $$\begin{array}{cc} & {\bar{T}}_{C}<{\bar{T}}_{C^{\prime }}⟺\\ & p_{c_{\alpha_{m}}}q_{c_{\alpha_{m}}}T_{c_{\alpha_{m}}}+p_{c_{\alpha_{m+1}}}q_{c_{\alpha_{m+1}}}T_{c_{\alpha_{m+1}}}<\\ & \;\;p_{c_{\alpha_{m}}^{\prime }}q_{c_{\alpha_{m}}^{\prime }}T_{c_{\alpha_{m}}^{\prime }}+p_{c_{\alpha_{m+1}}^{\prime }}q_{c_{\alpha_{m+1}}^{\prime }}T_{c_{\alpha_{m+1}}^{\prime }} \end{array}$$

By substituting Equations (2) and (8) in Equation (10), and by using again Equations (5) and (7), with some algebraic manipulations, it results:

(11) $$\begin{array}{cc} & {\bar{T}}_{C}<{\bar{T}}_{C^{\prime }}⟺p_{c_{\alpha_{m}}}q_{c_{\alpha_{m}}}T_{c_{\alpha_{m}}}+p_{c_{\alpha_{m+1}}}{\bar{p}}_{c_{\alpha_{m}}}q_{c_{\alpha_{m}}}T_{c_{\alpha_{m+1}}}<\\ & \;\;p_{c_{\alpha_{m+1}}}q_{c_{\alpha_{m}}}T_{c_{\alpha_{m+1}}}+p_{c_{\alpha_{m}}}{\bar{p}}_{c_{\alpha_{m+1}}}q_{c_{\alpha_{m}}}T_{c_{\alpha_{m}}}⟺\\ & p_{c_{\alpha_{m+1}}}p_{c_{\alpha_{m}}}q_{c_{\alpha_{m}}}T_{c_{\alpha_{m}}}<p_{c_{\alpha_{m+1}}}p_{c_{\alpha_{m}}}q_{c_{\alpha_{m}}}T_{c_{\alpha_{m+1}}} \end{array}$$

Equation (11) constitutes an absurdum since for hypothesis $T_{c_{\alpha_{m}}}>T_{c_{\alpha_{m+1}}}$.

By exploiting this result, it follows that to obtain the optimal channel set $C^{*}$, *i.e.*, the channel set that maximizes the expected throughput, the channels must be sorted according to their expected throughput, *i.e.*,

(12) $$C^{*}=\left\{\left(c_{\alpha_{1}}^{*},c_{\alpha_{2}}^{*},\ldots ,c_{\alpha_{N}}^{*}\right)\right):T_{c_{\alpha_{i}}^{*}}\geq Tc_{\alpha_{i+1}}^{*}\}$$

Equation (12) constitutes also a computational-efficient algorithm for evaluating the maximum expected throughput ${\bar{T}}^{*}$. In fact, its time complexity is bounded by O(nlogn) due to the sort operation. From Equations (3) and (12), we finally obtain the expression of the maximum expected throughput ${\bar{T}}^{*}$:

(13) $${\bar{T}}^{*}=\sum_{i=1}^{N}p_{c_{\alpha_{i}}^{*}}q_{c_{\alpha_{i}}^{*}}T_{c_{\alpha_{i}}^{*}}$$

## 5. Numerical Results

In this section, we adopt, as case study, a sensor network operating in the TVWS spectrum according to the IEEE 802.11af standard. This standard, also referred to as White-Fi, allows wireless local area network operation in TV white space spectrum in the VHF and UHF bands between 54 and 790 MHz.

First, we validate Equation (12) by showing that the expected throughput derived in Equation (13) constitutes the maximum expected throughput. The set of parameters is as follows. The TVWS spectrum is organized in N=5 channels, and the corresponding interference probabilities are uniformly distributed in [0,1]. By adopting a 6MHz bandwidth for the IEEE 802.11af standard, the set of achievable data rates is {0,1.8,3.6,5.4,7.2,10.8,14.4,16.2,18,21.6,24}Mbit/s, and the channel throughputs are uniformly distributed within the set.

**Figure 1:**
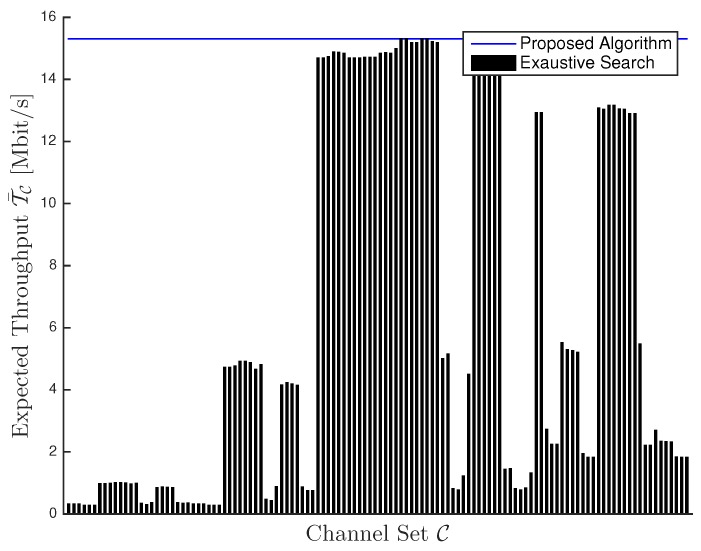
Expected Throughput ${\bar{T}}_{C}$: exhaustive search versus proposed algorithm.

Figure 1 shows the expected throughput ${\bar{T}}_{C}$ for each of the N!=120 sets {C}. Furthermore, we report the expected throughput derived in Equation (13). First, we note that expected throughput derived in Equation (13) effectively constitutes the maximum expected throughput. Hence, Equation (12) provides the sorting rule maximizing the expected throughput. Furthermore, we note that there exists a significant variability in terms of expected throughput among the different channel sets, ranging from less than 1 Mbit/s to over 15 Mbit/s. This result highlights the importance of studying the throughput achievable by an unlicensed sensor network operating over TV white space spectrum in presence of coexistence interference.

**Figure 2:**
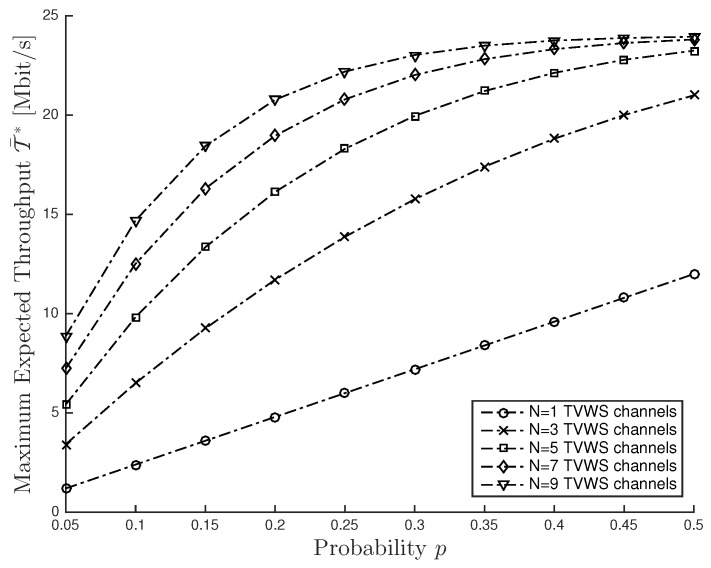
Maximum Expected Throughput ${\bar{T}}^{*}$ versus probability *p* for different number *N* of TVWS channels.

To better characterize the effects of the parameters on the expected throughput, in Figure 2 we show the maximum expected throughput ${\bar{T}}^{*}$ as a function of the channel availability probability for different values of the number *N* of TVWS channels, *i.e.*, N={1,3,5,7,9}. The simulation set is as follows: $p_{i}=p$ and $T_{i}=24$ Mbps for any i∈N. We can observe that the maximum average throughput is significantly affected not only by the probability *p*, but also by *N*. Specifically, we observe that, for the lower values of *p*, there exists a difference in terms of ${\bar{T}}^{*}$ for different values of *N*. In contrast, for the larger values of *p*, the difference in terms of ${\bar{T}}^{*}$ for different values of *N* decreases as *N* increases. Furthermore, we observe that the maximum expected throughput increases as *N* increases. This effect is reasonable, since *N* controls the degrees of freedom in terms of channel opportunities.

The conducted analysis has proved that the throughput available at an arbitrary secondary sensor network operating over shared TVWS space in presence of coexistence interference depends on the channel set. Furthermore, it has shown that there exists a computational-efficient algorithm for determining the channel set maximizing the throughput. Hence, in a nutshell, secondary sensor networks can maximize the throughput by scanning the channels in decreasing order of expected throughput and by utilizing the first channel not affected by coexistence interference.

## 6. Conclusions

In this letter, we studied the throughput achievable by an unlicensed sensor network operating over TV white space spectrum in presence of coexistence interference. Through the letter, first we analytically derived the achievable expected throughput as a function of the channel ordering, by showing that the derivation of the maximum expected throughput through exhaustive search is computationally unfeasible. Then we designed a computational-efficient algorithm with polynomial-time complexity for evaluating the maximum expected throughput. Finally, numerical results validated the theoretical analysis

## References

[1] Federal Communications Commission (2012). Second Memorandum Opinion and Order in the Matter of Unlicensed Operation in the TV Broadcast Bands.

[2] Ofcom (2012). Regulatory Requirements for White Space Devices in the UHF TV Band.

[3] Electronic Communications Committee (2013). Technical and Operational Requirements for the Operation of White Spaces Devices under Geo-Location Approach.

[4] Cacciapuoti A.S., Caleffi M., Paura L. Widely Linear Cooperative Spectrum Sensing for Cognitive Radio Networks. Proceedings of the Global Telecommunications Conference (GLOBECOM).

[5] Nguyen T., Koo I. (2015). Throughput Maximization for Sensor-Aided Cognitive Radio Networks with Continuous Energy Arrivals. Sensors.

[6] Shi Z., Wu Z., Yin Z., Cheng Q. (2015). Novel Spectrum Sensing Algorithms for OFDM Cognitive Radio Networks. Sensors.

[7] Wu C., Ohzahata S., Kato T. Dynamic channel assignment and routing for cognitive sensor networks. Proceedings of the International Symposium on Wireless Communication Systems (ISWCS).

[8] Cacciapuoti A.S., Caleffi M., Paura L. (2016). Optimal Strategy Design for Enabling the Coexistence of Heterogeneous Networks in TV White Space. IEEE Trans. Vehic. Technol..

[9] Cacciapuoti A.S., Caleffi M. (2015). Interference analysis for secondary coexistence in tv white space?. IEEE Commun. Lett..

[10] IEEE 802.22 Wireless Regional Area Networks Working Group (2011). 802.22-2011: Part 22: Cognitive Wireless RAN Medium Access Control (MAC) and Physical Layer (PHY) Specifications: Policies and Procedures for Operation in the TV Bands.

[11] IEEE 802.11 Wireless Local Area Network Working Group (2013). 802.11af-2013: Part 11: Wireless LAN Medium Access Control (MAC) and Physical Layer (PHY) Specifications Amendment 5: Television White Spaces (TVWS) Operation.

[12] IEEE 802.19 Wireless Coexistence Working Group (2014). 802.19.1-2014: Part 19: TV White Space Coexistence Methods.

